# Exploration of the associations between muscle oxygen saturation and skin temperature responses during isokinetic strength exercise

**DOI:** 10.1371/journal.pone.0349644

**Published:** 2026-06-22

**Authors:** Willian da Silva, Carlos Sendra-Pérez, Joaquín Martín Marzano-Felisatti, Inmaculada Aparicio-Aparicio, Felipe P. Carpes, Jose Ignacio Priego-Quesada

**Affiliations:** 1 Escuela de Kinesiología, Facultad de Ciencias, Pontificia Universidad Católica de Valparaíso, Valparaíso, Chile; 2 Research Group in Sports Biomechanics (GIBD), Department of Physical Education and Sports, Universitat de València, Valencia, Spain; 3 Department of Education and Specific Didactics, Jaume I University, Castellon, Spain; 4 Applied Neuromechanics Group, Laboratory of Neuromechanics, Federal University of Pampa, Uruguaiana, Brazil; Ordu University, TÜRKIYE

## Abstract

Previous studies suggested that skin blood flow, and therefore skin temperature affect changes in muscle oxygenation (SmO_2_). Given that near-infrared spectroscopy (NIRS) measurements consider a depth of a few centimeters to capture changes in SmO_2_, in this study, we aimed to explore whether a relationship between skin temperature and SmO_2_ is observed after sets of isokinetic strength exercises. Seventeen males (age: 25 ± 6 years old and body mass: 74.7 ± 12.4 kg) completed five series of 30 maximum unilateral (preferred) knee extension isokinetic exercises at 180º·sec^-1^ with a 60-second rest between series to exercise quadriceps muscles. At baseline, after a warm-up, and 50 seconds after each series of exercise, SmO_2_ related to vastus lateralis and skin temperature of the anterior thigh were evaluated in the exercised and non-exercised legs. Significance was established at p < 0.05. Exercise reduced mean skin temperature throughout the consecutive repetitions in both the exercised and non-exercised legs (p < 0.01). SmO_2_ increased during exercise only in the exercised leg. No significant correlations were found between skin temperature and SmO_2_, and weak correlations were observed between skin temperature and total haemoglobin for absolute (r = 0.4 and p < 0.001) and delta values (r = 0.3 and p = 0.03) only in the non-exercised leg. In conclusion, this exploratory study suggests that skin temperature and SmO_2_ are compatible as they explain different physiological responses, although these measurements are not associated during a controlled exercise.

## Introduction

Near-infrared spectroscopy (NIRS) devices provide information about muscle oxygen saturation (SmO_2_) by measuring changes in the balance between oxygen delivery and uptake in the muscle [1–3]. Portable NIRS devices have become increasingly popular in sports sciences to monitor skeletal muscle oxygen consumption during exercise [3]. Their applications include internal load monitoring [4,5], tracking microvascular function throughout rehabilitation [6,7], and determining metabolic thresholds [8], among others. However, measurement protocols still vary across studies, and sources of variability in the outcomes, such as skin blood flow [9–11], require further clarification.

Possible fluctuations in skin blood flow can introduce confounding errors in NIRS measurements [1]. Although contemporary NIRS devices algorithmically reduce the contribution of superficial tissues (e.g., adipose tissue), the technique detects signals from a depth of a few centimeters beneath the skin, and can still be influenced by skin blood flow in superficial veins, particularly when perfusion or temperature change [11]. Koga et al. [9] observed that whole-body heating increased both the absorption and scattering coefficients in NIRS signals, thereby altering the derived oxygenation parameters when optical factors were assumed to remain constant. Additionally, significant changes in skin blood flow, often associated with high or low skin temperatures, have been identified as potential confounding factors in SmO_2_ assessments [11]. These alterations in skin blood flow and temperature are common during exercise [12,13], highlighting the importance of understanding the specific effects of skin blood flow on the interpretation of NIRS-derived muscle oxygenation during exercise.

The methods for clarifying these potential influences are complex, as measuring skin vascularization is difficult in sports research due to the cost and complexity of laser Doppler devices. In contrast, infrared thermography (IRT) is a technique increasingly applied in the field, as it allows skin temperature to be measured [14,15]. Skin temperature is known to be closely linked to skin blood flow [16,17], as convective heat transfer through the circulation allows dissipation of the heat produced by elevated metabolic activity [18,19]. Although the combined use of both IRT and NIRS in the field was recently highlighted in an editorial [20], studies that investigate the theoretical relationship between skin temperature and SmO_2_ outcomes are lacking. It remains a challenge to determine whether the observed relationship is due to measurement errors in NIRS caused by skin blood flow or if it reflects an intrinsic connection between the two variables. For instance, an inverse correlation between skin temperature and SmO_2_ could occur during strength exercises, as a decrease in SmO_2_ [21] and an increase in skin temperature can happen [12]. Skin temperature is altered by exercise but exhibits slower post-exercise changes. At the same time, SmO_2_ rapidly recovers due to fast reperfusion, returning to baseline or slightly above and stabilizing between 30 seconds and 2 minutes, depending on the exercise performed [11,21,22]. For this reason, a possible approach to address this is to take measurements after completing a strength exercise and when SmO_2_ has been stabilized.

We consider that strength exercises with varying torque demand and movement amplitude may introduce confounding factors in SmO_2_ measurements. Stricter control could be achieved by testing strength exercises under isokinetic conditions. Therefore, we aimed to explore whether a relationship exists between skin temperature and SmO_2_ immediately after several series of strength exercises in an isokinetic configuration. Since we extracted the SmO_2_ value after the series and reperfusion were stabilized, we expected any association with changes in skin temperature to be weak.

## Materials and methods

### Design

To test the hypothesis that skin temperature is not a confounding factor of SmO_2_ measurements during exercise, we designed an experimental exploratory approach involving repeated knee extension exercises and simultaneous measurements of both variables for a correlational analysis. The study focused on SmO_2_ in the vastus lateralis muscle, as it is a primary contributor to knee extension and accessible for NIRS evaluation, ensuring reliable SmO_2_ measurements. Skin temperature of the anterior thigh was chosen as it reflects skin blood flow changes, providing complementary information on SmO_2_. Skin temperature was measured using infrared thermography for its distance characteristic that does not interfere with the heat dissipation of the region, as happens with thermal contact sensors and its method of attachment that reduces heat dissipation by radiation and convection [23,24]. The isokinetic exercise protocol at 180º·sec ⁻ ¹ was chosen to ensure consistent muscle recruitment across repetitions and minimize variability. Measurements were taken at baseline (before a standardized warm-up) and 50 seconds after each series of isokinetic knee extension contractions to capture dynamic changes in skin temperature and SmO_2_, while avoiding the effect of reperfusion of SmO2 after exercise and the possible correlation while exercising when both variables can be correlated but are not related with the potential confounding effect.

### Participants

Seventeen physically active (performing at least two strength training sessions per week), healthy (without disease or injury condition diagnosed) and Caucasians, recruited from the local community (university students invited by email to be part of the study) were included (mean ± standard deviation, age: 25 ± 6 years old; body mass: 74.7 ± 12.4 kg; height: 1.76 ± 0.0 cm; body mass index: 23.99 ± 2.67 kg/m^2^; skinfold thickness: (right thigh) 19.39 ± 9.38 mm and (left thigh) 20.33 ± 9.90 mm). The right was the preferred leg by all participants except one. Exclusion criteria also included smokers and participants who ingest medications or nutritional supplements.

After recruiting 17 participants, we performed a post hoc power analysis to evaluate the adequacy of the sample size. Assuming a potential correlation of r = 0.20 between skin temperature and SmO_2_, with a type I error rate (α) of 0.05 and a type II error rate (β) of 0.20 (power = 80%), the required sample size was estimated to be 194 participants [25]. Given that such a weak correlation would have limited physiological relevance and that further recruitment would have imposed disproportionate costs, data collection was stopped after 17 participants. The recruitment period for this study started on March 10^th^ 2023, and finished on June 20^th^ 2023. All participants signed a consent form before enrolling in the study. All procedures complied with the Helsinki Declaration, and the Ethics Committee of Research in Humans of the University of Valencia approved this research (IRB #2626957).

The experimental design consisted of a single visit to the laboratory. First, anthropometric data were collected, which included body composition variables obtained using a bioelectrical impedance system (Tanita BC-545N, Tanita Corp., Tokyo, Japan). Anterior thigh skinfold was measured for both lower limbs (mean of three measurements) by the same researcher using a calliper (Boz-Calpro-3, Bozeera, China) following the protocol for anthropometric assessment of the International Society for the Advancement of Kinanthropometry (ISAK). Measurements were performed by a researcher with more than 10 years of experience performing anthropometric assessments and holding a ISAK Level 1 certification. Then, participants completed an isokinetic exercise protocol while SmO_2_ and skin temperature were measured. Participants were requested to refrain from exercise for 24 hours before testing and to maintain their usual hydration and nutrition habits, but to have their last meal at least two hours before the visit to the laboratory. In the 48 hours previous to testing, participants were instructed to avoid sunbathing or using creams or cosmetics on their skin in order to prevent these factors having an effect on the measurements [26]. Moreover, they were asked to refrain from consuming alcohol, coffee or any stimulant drink for 6 hours before the visit.

**Fig 1:**
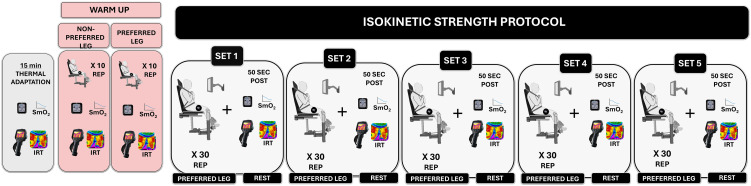
Experimental design. SmO_2_: Muscle oxygen saturation; IRT: Infrared thermography.

**Fig 2:**
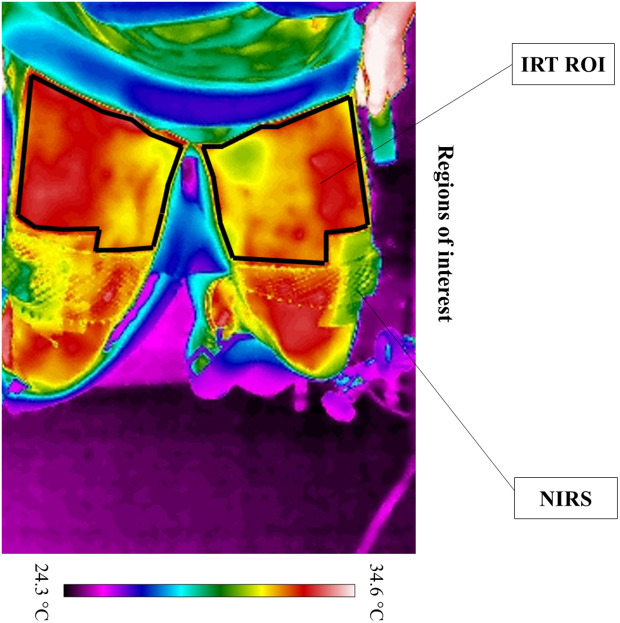
Representation of the regions of interest (ROIs) considered for skin temperature analyses. This image is representative of all participants. IRT: Infrared thermography. NIRS: Near-infrared spectroscopy.

For placement of the NIRS device (see item 2.3), each participant was instructed to lay on a stretcher. The determination of the two regions of interest (ROIs) (Fig 2) for future IRT analyses was made a posteriori. SmO_2_ and skin temperature measurements were performed before and after 50 seconds of each series of the exercise protocol. Fig 1 depicts the experimental design.

### Procedures

The exercise protocol was designed to elicit acute muscle responses of the quadriceps muscles through the repetitions of unilateral isokinetic knee extension contractions using an isokinetic dynamometer (Biodex Multi-joint System Pro 3, Shirley, NY, USA). Participants were adjusted to the dynamometer seat to have the dynamometer’s axis aligned with the knee joint axis (i.e., line determined between condyles lateral and medial of the femur). The distal portion of the lower limb was attached to the lever of the dynamometer 3 cm above the lateral malleolus of the fibula [27].

The participants were instructed to keep their arms crossed over their chest. Furthermore, belts were used around the waist, torso, and contralateral thigh to minimize compensatory movements. The lower limb was weighted to remove the limb’s gravitational effect for each trial [27]. The exercise protocol began with the participants completing a warm-up (10 knee extensions without load) in both legs (Fig 1). After a 1-minute interval, the exercise protocol started for the preferred leg.

The protocol was performed only with the preferred leg and involved five series of 30 repetitions of concentric knee extension at 180 º·sec^-1^ with a 60 seconds rest between series [28]. Each contraction began with the knee flexed at 90º and continued until the knee was fully extended. During the test, participants were verbally encouraged to produce maximal strength during all repetitions.

The SmO_2_ and total haemoglobin (THb) were continuously sampled at 0.5 Hz bilaterally in the muscles vastus lateralis (i.e., preferred and non-preferred legs) using a Moxy Monitor (Fortiori Design LLC, Minneapolis). This device has an emitter-detector spacing of 25 mm and a penetration depth of 12.5 mm, validated by a previous study [29]. Devices were positioned over the belly of the vastus lateralis muscle at 2/3 of the distance from the anterior superior iliac spine to the lateral border of the patella, using the SENIAM guidelines for spatial reference and standardization across participants [30]. Before attaching the devices, the area underwent trichotomy and was cleaned with alcohol for proper fixation.

After visual inspection of all the individual signals, SmO_2_ values (a mean of 5 seconds) were obtained at baseline before the exercise, after the warm-up, and 50 seconds after each series of exercise, coinciding with the same time that IRT images were acquired. A time of 50 seconds was set as it was observed during experimental pilot studies that it is at this measurement timepoint when reperfusion of SmO_2_ stabilizes. THb values were also obtained (arbitrary units). Delta values (the difference between each measurement timepoint and baseline) were also calculated.

The skin temperature was measured using an IRT camera (E54 model, 320 x 240 pixels resolution, NETD <40 mK at 30º, and measurement uncertainty of ± 2°C or 2% of the reading, Flir Systems Inc., Wilsonville, USA). The camera was turned on for 10 minutes before all evaluations to allow adequate stabilization of the components, and all participants completed a thermal room adaptation of 15 minutes for baseline measures [31]. During this period, they were instructed to remain seated in the dynamometer and not perform any movement or touch the skin of the ROIs.

The ROIs corresponded to the proximal third of the thigh, located above the Moxy sensor up to the line between the groin area and external portion of the anterior thigh, in the preferred and non-preferred legs (Fig 2). The thermographic camera was positioned perpendicular to the ROIs, and the same evaluator captured all the images. Images were acquired at baseline, after warming up, and 50 seconds after each series of 30 repetitions of isokinetic knee extension contractions. ROIs were uncovered by clothing during the whole experiment. The mean and standard deviation environmental conditions: Room temperature was 22.3 ± 2.8°C, and air humidity was 49.9 ± 5.0%. All IRT procedures followed the Thermographic Imaging in Sports and Exercise Medicine checklist [32].

The mean of the skin temperature was obtained for the ROIs using an emissivity of 0.98 with the ThermaCAM Researcher Pro software (version 2.10, FLIR, Wilsonville, USA). During the exercise protocol, we also determined the skin temperature of the anterior thigh that was not performing knee extension as a control measure. Absolute and delta (the difference between each measurement timepoint and baseline) skin temperature values were also obtained.

### Statistical analyses

Statistical analyses were performed using RStudio (version 2024.09.0). Data distribution was checked using Shapiro-Wilk. A parametric distribution was found for skin temperature and THb, while SmO_2_ data showed a non-parametric distribution. Therefore, to compare the skin temperature and THb (both absolute and delta values), repeated measures ANOVAs with two factors (limb and protocol measurement timepoint) and Bonferroni post-hocs. For SmO_2_ data, Friedman tests with Wilcoxon post-hocs with Bonferroni correction were applied. The 95% confidence interval (95%CI) of the differences was obtained, and Hedge’s effect sizes (ESg) were computed and classified as small (ESg 0.2–0.5), moderate (Esg 0.5–0.8), or large (ESg > 0.8). A linear regression analysis was performed, including data on skin temperature and SmO_2_, and skin temperature and THb, for absolute and delta values. Bivariate Pearson correlation coefficients (r), adjusted R^2^, p-values with Bonferroni correction (across the six comparisons evaluating the association between skin temperature and SmO₂, and the six comparisons between skin temperature and THb), and equations of the adjustments were obtained. Significant correlations (p < 0.05) were classified as weak (0.2 < |r| < 0.5), moderate (0.5 ≤ |r| < 0.8), or strong (|r| ≥ 0.8) [33]. The significance level was set at 0.05.

## Results

**Fig 3:**
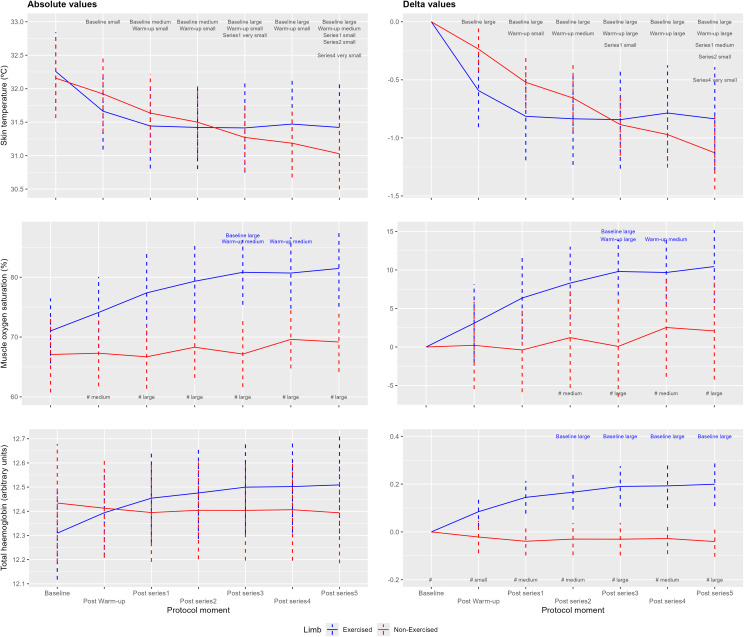
Mean (line) and confidence interval at 95% (dashed lines) of the skin temperature, muscle oxygen saturation, and the total haemoglobin response during the limb exercise and non-exercised protocol. Differences between protocol measurement timepoint were referenced at each timepoint with the magnitude of the effect size (grey letters for the main effect for both limbs and blue letters for the exercised limb). Differences between limbs were shown by # (p < 0.05) with the magnitude of the effect size.

Both absolute and delta values of skin temperature showed a main effect for protocol measurement timepoint (absolute F_(6, 182)_=3.1, p < 0.01; delta F_(6, 182)_=11.1, p < 0.001, Fig 3), without main effects for the limb (absolute F_(1, 182)_=0.2, p = 0.70; delta F_(1, 182)_=0.3, p = 0.57, Fig 3) or interaction between protocol measurement timepoint and limb (absolute F_(6, 182)_=0.4, p = 0.89; delta F_(6, 182)_=1.4, p = 0.21, Fig 3). As similar differences were observed between measurement timepoints in absolute and delta values, only differences of deltas are reported in the text. Skin temperature decreased as the exercise protocol progressed, especially in relation to the baseline (e.g., Baseline vs. Post series 5, mean difference 1.0ºC and 95 CI%[0.7, 1.2ºC] p < 0.001 and ESg = 2.04).

No differences between limbs were observed at baseline for SmO_2_ (p = 0.06). Only the exercised limb presented an increase in SmO_2_ during the protocol, showing differences at the Post Series 3 with the Baseline (mean difference 10.6% and 95 CI%[6.3, 15.4%] p = 0.04 and ESg = 1.88) and the Warm-up (mean difference 6.4% and 95 CI%[4.1, 9.4%] p < 0.01 and ESg = 0.81), and the Post Series 4 with the Warm-up (mean difference 7.0% and 95 CI%[4.0, 10.4%] p = 0.03 and ESg = 0.78). As the protocol progressed, the differences between both legs became greater (e.g., at Post Series 5: absolute values: mean difference 14.9% and 95 CI%[7.7, 19.5%] p = 0.01 and ESg = 1.20, delta values: mean difference 11.2% and 95 CI%[5.4, 15.3%] p = 0.001 and ESg = 0.84).

THb showed no main effect for protocol measurement timepoint (F_(6, 182)_=0.2, p = 0.97), limb (F_(1, 182)_=0.71, p = 0.4) or interaction between protocol measurement timepoint and limb (F_(6, 182)_=0.4, p = 0.87). However, although delta THb showed no main effect for protocol measurement timepoint (F_(6, 182)_=1.9, p = 0.08), there was a main effect of limb (F_(1, 182)_=99.2, p < 0.001), and interaction between protocol measurement timepoint and limb (F_(6, 182)_=3.7, p < 0.01). THb was lower at baseline than at the different post series for the exercised lower limb (e.g., Baseline vs. Post series 5: mean difference 0.2 arbitrary units and 95 CI%[0.1, 0.3 arbitrary units] p < 0.01 and ESg = 1.8; exercised vs. non-exercised at Post series 5: mean difference 0.3 arbitrary units and 95 CI%[0.1, 0.4 g arbitrary units] p < 0.001 and ESg = 0.8).

**Fig 4:**
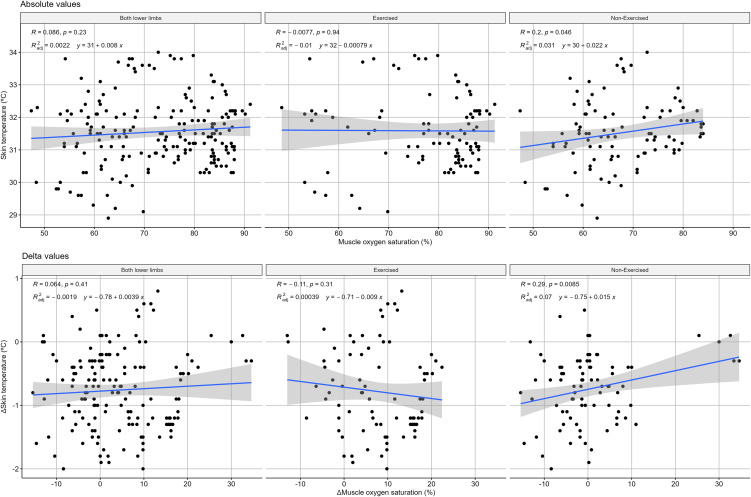
Linear regression analysis between absolute and delta (∆) values between the skin temperature and the muscle oxygen saturation values.

Considering pooled data from lower limbs and data from each limb (exercised and non-exercised), no significant correlations were found between skin temperature and SmO_2_ for absolute (both lower limbs p = 1.00; exercised limb p = 1.00; and non-exercised limb p = 0.27) and delta values (both lower limbs p = 1.00; exercised limb p = 1.00; and non-exercised limb p = 0.05, Fig 4).

**Fig 5:**
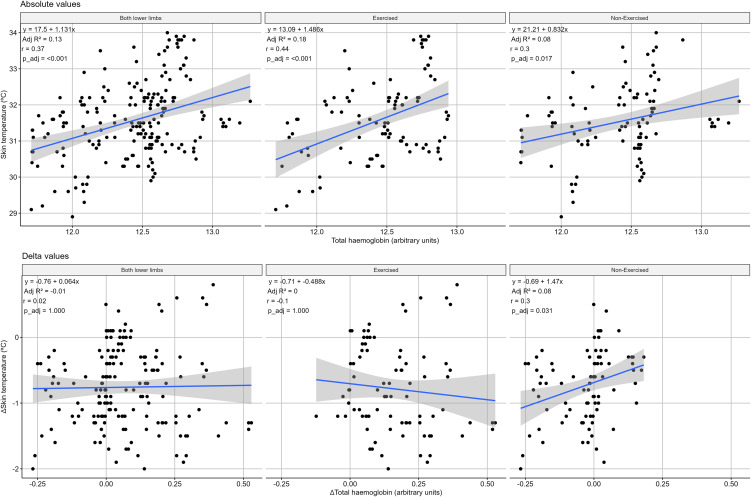
Linear regression analysis between absolute and delta (∆) values between the skin temperature and the total haemoglobin values.

Skin temperature and THb presented weak correlations for both limbs (Fig 5, exercised: r = 0.4 and adjusted R^2^ = 0.18, 95 CI%[0.3, 0.6], and p < 0.001; non-exercised: r = 0.3 and adjusted R^2^ = 0.08, 95 CI%[0.1, 0.5], and p < 0.001). For delta values, only the non-exercised limb presented weak correlations between skin temperature and THb (r = 0.3 and adjusted R^2^ = 0.08, 95 CI%[0.1, 0.5], and p = 0.03).

## Discussion

In this exploratory study, our aim was to assess whether a relationship exists between skin temperature and variables measured by NIRS technology during isokinetic strength exercises (at rest, 50 seconds after each series of isokinetic knee extension contractions). Our main findings support our hypothesis that NIRS variables (i.e., THb and SmO_2_) could either not correlate or show only a weak direct correlation with exercise-induced changes skin temperature post-exercise. Based on our experimental design, the potential error in NIRS evaluation caused by changes in skin temperature appears to be minimal in a moderately controlled environmental scenario. Furthermore, caution is needed when associating fluctuations in skin temperature as determinants of short-time acute changes in SmO_2_.

The changes in skin blood flow induced by interventions such as thermal stress tests were previously suggested as potential confounding factors in SmO_2_ assessments [11]. However, technologies and their algorithms have advanced, and it is now worth investigating limitation in this method. Moreover, previous studies found that skin temperature relates to skin blood flow [16,17]. However, we found no significant correlations between both outcomes in the exercised and non-exercised lower limb during a controlled exercise condition. This lack of association may reflect the different physiological mechanisms underlying both variables: while skin temperature mainly reflects peripheral thermoregulatory responses mediated by cutaneous blood flow, SmO₂ primarily reflects local muscle oxygen extraction. In addition, SmO₂ was extracted once reperfusion had stabilized after the exercise series, which may have further reduced any transient coupling between peripheral temperature responses and muscle oxygenation.

There is a lack of studies that describe associations between SmO_2_ or THb and skin temperature during exercise. Hom et al. [34] observed that pre-warming the skin elicits lower decreases in SmO_2_ during an isotonic forearm exercise, while pre-cooling amplifies this decrease. These findings suggest that skin temperature may modulate oxygen dynamics, particularly under moderate exercise intensity and environmental conditions. However, the main limitation of these studies was using thermocouples to obtain temperature data. Thermocouples and infrared thermography data may not always agree [23,24]. Moreover, the different ROI measured (forearm vs. vastus lateralis) and the different scale of the responses observed in skin temperature (~7.7 after cooling *vs.* ~ 1.5°C after exercise) make comparisons between both types of studies difficult. Regarding this point, it is important to note that our results are applicable to a moderate environmental scenario when changes in skin temperature are not extreme. Under these controlled conditions, peripheral thermal responses may remain relatively small and independent from the local oxygenation dynamics measured in the muscle. This may partly explain why no significant correlations were observed between skin temperature and SmO₂ in either the exercised or non-exercised limb.

In addition, in line with Cum et al. [35], our results showed a weak correlation between THb and skin temperature, similar to what occurs with other physiological variables (i.e., VO_2_ and heart rate) during the incremental test. However, it should be noted that THb values are lower than hemoglobin concentrations measured in blood by venous sampling [36]. Although the observed correlations should be interpreted with caution due to their low magnitude, a possible explanation is that both variables are partially influenced by changes in peripheral blood flow. For instance, peripheral vasoconstriction during exercise may reduce cutaneous blood flow and skin temperature while simultaneously affecting local blood volume in the muscle microvasculature. Conversely, increased peripheral vasodilation under greater thermal stress may promote both higher skin temperatures and increased local blood volume, which could be reflected in THb measurements.

Unlike previous studies, our exercise protocol might be one of the first designed to control confounding factors related to range of motion, velocity, and intensity of the exercise. We considered a protocol of 5 series with 30 repetitions each for knee isokinetic extension at maximum intensity to elicit a maximal exercise intensity. Also, we acquired SmO_2_ data after the stabilization of the reperfusion and not during exercise (i.e., at the same time when IRT images were captured), which may reduce the influence of artifacts in the SmO_2_ outcomes. For this reason, as previous studies showed [11,21,37], after finishing the exercise, reperfusion increased until it reached similar or higher values of SmO_2_, which explains the increase of SmO_2_ values in our data, and the absence of responses of the non-exercised lower limb.

Based on previous studies [38,39], an increase in skin temperature during strength exercise could be expected. However, we found that skin temperature decreased during the exercise protocol. Skin temperature responses during exercise could be explained by the result of the heat transferred by the skin blood flow, heat dissipation processes (conduction, convection, radiation and sweat evaporation), and biophysical factors (e.g., body composition or body surface area) [19,40]. The lack of increases in skin temperature in our participants may result from the heat generated in the muscle being not transferred by conduction, since this process is considered slow when there is no significant thermal gradient between the muscle and the skin [19]. Therefore, the decrease in skin temperature observed is likely to be the result of peripheral vasoconstriction aiming to prioritize blood flow to the exercised muscled [41]. This assumption is in agreement with the hypothesized in previous studies [42,43]. These differing responses to both outcomes underline the fact that skin temperature and SmO₂ reflect distinct physiological mechanisms. While skin temperature mainly reflects cutaneous blood flow and heat exchange with the environment, which can also be influenced by internal heat production from muscular work, SmO₂, primarily represents oxygen utilization within the muscle tissue.

Koga et al. [44] compared SmO₂ and muscle activation by surface electromyography, and observed that the results should be interpreted with caution, as there is certain heterogeneity depending on the fibers being measured (especially with the NIRS device, which measures a small region). The region assessed for both devices (NIRS and IRT), in terms of location and size, also deserves attention. It is impossible to evaluate both signals at the same location as NIRS covers the measurement area. However, while SmO_2_ is typically measured on a small area of a muscle, skin temperature usually covers a large area (i.e., the entire superficial region of the muscle). Moreover, although SmO_2_ could be affected by small changes in location [44], a previous study showed that there are no significant differences in the skin response to exercise of different segments of the anterior thigh [45], which suggests that our measured region correctly characterizes the anterior thigh skin temperature response.

The findings of this exploratory study provide valuable insights for coaches, sports scientists, and practitioners utilizing NIRS and IRT to monitor physiological responses during exercise in a moderate environmental scenario that might be useful for physical testing of participants, as well as to monitor short and long-term adaptation to training programs. Our results suggest that while skin temperature and SmO_2_ or THb are complementary measurements of different physiological processes, they are not directly associated during controlled strength exercises. This distinction highlights the importance of interpreting these measurements independently, particularly when assessing muscle oxygenation and peripheral blood flow adaptations. For practitioners, this means that fluctuations in skin temperature should not be used as a proxy for acute changes in SmO_2_ during or immediately after exercise.

Sample size limitation should be considered when interpreting the results, as the study may not have been sufficiently powered to detect small associations between variables. However, small magnitude associations might not reach clinically significant in this context. Moreover, the absence of statistically significant correlations should be interpreted as lack of evidence for a meaningful association under the present conditions rather than definitive proof that no relationship exists. As we have assessed a physically active population, our hypothesis still needs to be tested with other populations with less adipose tissue (e.g., professional athletes) or higher adipose tissue (e.g., sedentary), as the level of skinfold thickness could increase the error of NIRS [46] and the transference of the heat from deep tissues to the skin [47]. Although it was suggested that skinfolds greater than 25 mm are critical for the NIRS error [48], in our study, all participants had fold values under these skinfold levels. Furthermore, we mitigated this error risk by using delta values [49]. The room temperature had a standard deviation of 2.8, higher than desired, however, the range of the room temperature was within the recommended range of 18–25ºC [50]. Although this may have increased the variability in skin temperature among participants, it is not thought to have affected their response during exercise and therefore not to have affected the primary objective of analyzing correlations between SmO_2_ and skin temperature.

In conclusion, the present exploratory study suggests that variables measured by NIRS technology and skin temperature may serve as complementary measures, reflecting distinct physiological responses; however, they are not associated during controlled exercise. While further studies are needed to cover broader characteristics of exercises, we advise caution when interpreting fluctuations in skin temperature as determinants of short-time acute changes in SmO_2_.

## Supporting information

S1 File Inclusivity-in-global-research-questionnaire. (DOCX)
